# Biobanking for health in Latin America: a call to action

**DOI:** 10.1016/j.lana.2024.100945

**Published:** 2024-11-11

**Authors:** J. Adrián Rivera-Alcántara, Carlos A. Aguilar-Salinas, Alexandro J. Martagon

**Affiliations:** a Escuela de Medicina y Ciencias de la Salud, Tecnologico de Monterrey, Mexico City, Mexico; b Unidad de Investigación de Enfermedades Metabólicas, Instituto Nacional de Ciencias Médicas y Nutrición Salvador Zubirán, Mexico City, Mexico; c The Institute for Obesity Research, Tecnologico de Monterrey, Monterrey, Mexico

Precision medicine aims to use individual characteristics to prevent, diagnose, and treat diseases more accurately and to refine the development of scales for disease prediction and risk stratification, leading to better health outcomes.^1^ Biobanking, the systematic collection and storage of biological samples and their associated data is a cornerstone for biomedical research and precision medicine.^2^ Moreover, biobanks enable large-scale epidemiological studies on genomic, environmental, and lifestyle factors influencing individual and population health.

Current world references in biobanking include the UK Biobank (UKB) and the All of Us Research Program (AoURP), from the US, along with large genomic datasets like the Genome Aggregation Database (global), which have significantly contributed to global research.^3^ Particularly, the AoURP is designed to include half of its sample from diverse ethnic groups in the United States and about 75% from underrepresented minorities.

Despite these efforts, most research findings continue to be predominantly based on White-European ancestry data, which may not apply entirely to other ethnicities.^4^^,^^5^ In addition, findings from genomic studies have shown high genetic diversity and disease heterogenicity in Latin American populations, requiring precise and tailored solutions.^6^, ^7^, ^8^ Therefore, dedicated biobanks for Latin America's underrepresented groups and diverse ethnicities are essential to improve and advance precision medicine.

The UK has developed a conducive environment through a regulatory framework, with independent but complementary regulations, that encompasses ethical management and storage, informed consent, privacy, and data protection.^3^ Although there are no dedicated laws for biobanking, the existing framework is sufficiently broad and precise to cover biobanking activities, providing flexibility and consistency across biomedical research. These regulations were established through collaboration between stakeholders, including the public, government, regulatory agencies, and academia. Following this example, Latin American countries could greatly benefit from similar collaboration to develop or adapt regulations that promote biobanking growth and development.

Using the UKB as a reference, countries like Brazil, Chile, and Mexico have advanced considerably in developing biobanks and precision medicine. These countries have established biobanks primarily focusing on cancer, metabolic, infectious, and neurological diseases research.^9^ Despite these advancements, biobanks in the region face significant obstacles, including insufficient funding, absent regulatory framework, and standardisation issues, which diminish their impact compared to their counterparts in Europe and North America.

Robust governance is essential for biobanks' success and sustainability.^10^ This concept refers to the interrelation between stakeholders, biobanking activities, and the legal framework. Most Latin American countries lack comprehensive biobanking regulations, allowing poor standardisation, impeding the recognition of biobanks as legal entities, and hindering biobanking activities and progress.

In Latin America, Brazil boasts the most robust system for biobanking and is the only country with dedicated regulations for biobanks. By addressing ethical, technical, and legal aspects, this regulatory framework has enabled biobank governance and convergence of efforts, creating large multicentric biobanks and international collaborations, which are translated into high-impact research. In contrast, the rest of the region still needs harmonised and unified biobank regulations. Some places, such as Mexico and Puerto Rico, already have some legal frameworks for genomic and biomedical research, however, there is no explicit mention of biobanks, and the current regulations do not entirely apply to biobanking. Contrary to the belief that fewer regulations make biobanking easier, the lack of specific regulations hinders the growth of biobanks, impedes collaboration, makes data security and quality inconsistent, and complicates ethical and legal issues.

The success of the UKB relies on robust governance, with the interplay between stakeholders, unified regulations, and legal and ethical frameworks being crucial. Consequently, rigorous scientific methods and adequate funding have led to approval from regulatory agencies. Similarly, in Mexico, the Mexican Biobank of Metabolic Diseases (BIOMEM) has recently gained approval from the Mexican Federal Commission for Protection against Health Risks (COFEPRIS), becoming the first legally recognised biobank in the country. This marks significant progress in regulating biobanks in the region and may serve as an example of collaboration between biobanks, government, and regulatory bodies towards standardising biobanking activities and regulations, fostering biobanks development in Latin America.

The evaluation, approval, and recognition of the BIOMEM were only possible through collaborative work between COFEPRIS and research, clinical and academic institutions. Nevertheless, an open and readily available process aligning every biobank initiative to a standardised legal framework is needed. With extended knowledge across international borders, we will be able to comprehend biobanking peculiarities and approach them accordingly.

We urge governments, research institutions, and international bodies to prioritize the development of biobanking regulations to unlock the full potential of precision medicine in Latin America. In critical times for healthcare systems, collaboration and awareness of the potential impact of our actions are decisive for our future.

## Contributors

JARA: Conceptualization, investigation, writing—original draft & editing.

AJM: Supervision, conceptualization, writing—original draft, review & editing.

CAAS: Supervision, writing—review & editing.

## Declaration of interests

The authors declare no competing interests. No funding source was required for this article.
